# Transarterial chemoembolization *vs.* liver resection as initial treatment for hepatocellular carcinoma occurring exclusively in caudate lobe: A retrospective propensity matching analysis

**DOI:** 10.32604/or.2022.026044

**Published:** 2022-12-06

**Authors:** BIAO YANG, MINLAN YUAN, TING YANG, ZHENGYIN LIAO, HAO WU

**Affiliations:** 1 Department of Abdominal Oncology, West China Hospital, Sichuan University, Chengdu, China; 2 Department of Psychiatry, West China Hospital, Sichuan University, Chengdu, China; 3 Department of Gastroentology, West China Hospital, Sichuan University, Chengdu, China

**Keywords:** Transarterial chemoembolization, Liver resection, Hepatocellular carcinoma, Caudate lobe

## Abstract

Treatment of hepatocellular carcinoma (HCC) in the caudate lobe is technically challenging. This retrospective study was designed to evaluate the clinical outcome of both superselective transcatheter arterial chemoembolization (TACE) and liver resection (LR) for HCC occurring exclusively in the caudate lobe. From January 2008 to September 2021, a total of 129 patients were diagnosed with HCC of the caudate lobe. The Cox proportional hazard model was used to analyze the potential clinical factors and established prognostic nomograms with interval validation. Of the total number of patients, 78 received TACE and 51 received LR. The overall survival (OS) rates (TACE *vs*. LR) at 1, 2, 3, 4, and 5 years were 83.9% *vs*. 71.0%; 74.2% *vs*. 61.3%; 58.1% *vs*. 48.4%; 45.2% *vs*. 45.2%; and 32.3% *vs*. 25.0%, respectively. However, subgroup analysis revealed that TACE was superior to LR for treating patients with stage IIb Chinese liver cancer (CNLC-IIb) in the entire cohort (*p* = 0.002). Interestingly, no difference was found between TACE and LR in the treatment outcomes of CNLC-IIa HCC (*p* = 0.6). Based on Child-Pugh A and B calculations, TACE tended to lead to a better OS than LR (*p* = 0.081 and 0.16, respectively). Multivariate analysis showed that Child-Pugh score, CNLC stage, ascites, alpha fetoprotein (AFP), tumor size, and anti-HCV are related to OS. Predictive nomograms for 1, 2, and 3 years were performed. Based on this study, TACE may provide a longer OS than liver resection for patients with CNLC-IIb HCC of the caudate lobe. Because this suggestion is limited by the study design and relatively small sample size, additional randomized controlled trials are needed.

## Introduction

Hepatocellular carcinoma (HCC) is the third leading cause of cancer-related death worldwide [1]. HCC arising in the hepatic caudate lobe is rare and has a poor prognosis [2,3]. Treatment for HCC originating in the caudate lobe is limited because the cancer cells are centrally located between the right and left lobes of the liver, near the hepatic hilum and the inferior vena cava [4,5]. Liver resection (LR) of the caudate lobe is considered a radical treatment. However, surgical resection represents an enormous technical challenge, and there is greater recurrence in the hepatic caudate lobe than in other lobes [6–9]. Similarly, both percutaneous ethanol injection and percutaneous radiofrequency ablation are potential treatments for caudate lobe HCC. However, these procedures are technically challenging, as the malignant lesions are deep and close to large blood vessels [10–12].

Transcatheter arterial chemoembolization (TACE) has been suggested for HCC treatment by the Barcelona Clinic Liver Cancer (BCLC) staging system, the European Association for the Study of the Liver, and Guidelines for the Diagnosis and Treatment of Primary Liver Cancer in China (2017 edition) [13–15]. Kim et al. [16] have presented a procedural technique for chemoembolization of HCC in the caudate lobe. These studies indicated that TACE is a candidate treatment for caudate lobe HCC. However, evidence supporting TACE’s application only to HCC originating in the caudate lobe is quite limited. Zhou et al. [17] reported that the 1- and 3-year overall survival rates after surgery were 90.1% and 60%, respectively. These results are similar to those from another study, where Terayama et al. [18] reported that the OS after embolization was 89% and 74% after 1 and 3 years, respectively. As of now, study has compared LR and TACE treatment for HCC occurring exclusively in the caudate lobe. We therefore conducted this retrospective study to compare the overall survival rates in patients treated with LR and TACE for HCC originating in the caudate lobe.

## Materials and Methods

### Study design

**Supplementary Figure S1:**
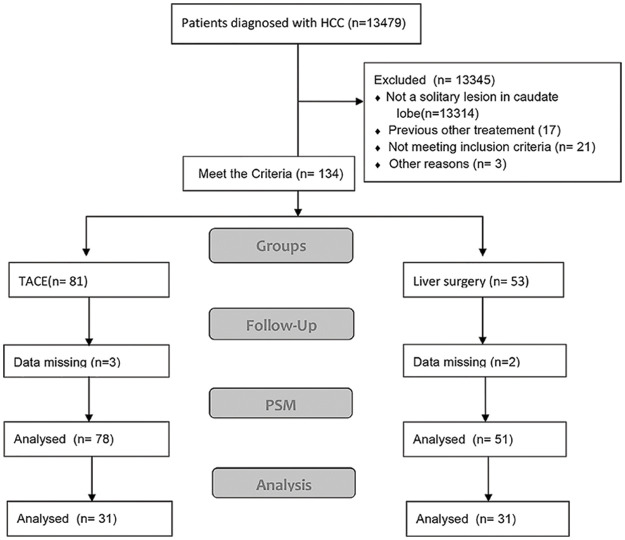
Flow diagram of patients included in this study.

Our local ethics committee approved this retrospective study. Written informed consent was obtained from each patient. From January 2008 to September 2021, a total of 7,748 patients with HCC received TACE and 5,731 received LR. Among these patients, 123 were diagnosed with HCC only in the caudate lobe; they were included in this study (TACE *vs*. LR; 78 *vs*. 51) (Suppl. Fig. S1).

### Eligibility criteria

Inclusion criteria:

(1) For patients in TACE group, HCC confirmed by pathology or at least two imaging scans (contrast-enhanced ultrasound, computed tomography (CT), or magnetic resonance imaging (MRI)) and serum AFP levels [15]; for patients in the LR group, HCC confirmed by pathologyHCC lesions occurring exclusively in the caudate lobe; (2) HCC lesions occurring exclusively in the caudate lobe; (3) Child-Pugh A/B liver function; (4) Eastern Cooperative Oncology Group performance (ECOG) status of below 2; (5) International normalized ratio below 1.5; (6) No history of any disease-specific treatment, including drugs or interventional treatment, in the previous six months; (7) No concurrent severe medical illness.

Exclusion criteria:

(1) Age 75 years or older or below 18 years; (2) History of esophageal variceal bleeding in the previous 3 months; (3) Having received systemic antitumor drug therapy; (4) Pregnancy; (5) History of hemoperitoneum due to acute tumor rupture; (6) Lesions in other lobes; (7) History of hepatic encephalopathy; (8) Presence of any other concurrent malignancy.

### Procedures

Before TACE, an enhanced CT scan was performed to confirm the nutrient vasculature of the tumor. This was done as previously described [16]. Briefly, the Seldinger technique under local anesthesia was used to access the femoral artery. A 5F-angiographic catheter was used to perform celiac or common hepatic arteriography via the right femoral artery this procedure was monitored by digital subtraction angiography (DSA). Once the arteries feeding the tumor had been identified by hepatic angiography, an emulsion of 5–15 mL of iodized oil and 40–50 mg of epirubicin was injected into the superselected supplementary artery until the artery was completely obstructed, and the tumor stain had disappeared from the DSA monitor. When tumor recurrence was identified by CT or MRI, angiography and TACE were repeated.

Single or multiple liver resections were performed to remove tumor in the caudate lobe. Before resection, the abdominal cavity was carefully searched for the extent of local lesions; then the blood flowing into the liver was occluded using Pringle’s maneuver. Additionally, a rubber tourniquet was applied to encircle and tighten the entire hepatoduodenal ligament. Finally, the clamp crushing method was used to resect the liver. Tumor clearance at the negative resection margins on frozen section of at least 5 mm was considered adequate to define the surgical procedures as curative [19].

### Follow-up and assessment

According to the classification system of Kumon et al. [5], the caudate lobe is partitioned into three subsegments: the Spiegel lobe, paracaval portion, and caudate process. If the tumor was large enough to occupy two or more subsegments, we defined the dominant one as the tumor-located subsegment. All patients were followed-up by the same multidisciplinary team after treatment. The HCC lesions were classified according to the China Liver Cancer Staging System (CNLC) [20]. OS was defined as the period from procedure to death or last follow-up. Adverse events were evaluated using the National Cancer Institute Common Terminology Criteria for Adverse Events, version 4.0 (CTCAE) [21]. Enhanced CT/MRI images were again obtained 4 weeks after the procedure to assess oil accumulation and response.

### Statistical analysis

SPSS 20 (IBM, New York, USA) and R-studio were used for statistical analysis, and continuous variables were expressed as mean with standard deviation (SD) or median (range). Categorical variables were compared by the Chi-square test or Fisher’s exact test and continuous variables by Student’s *t*-test. The Kaplan-Meier method was used to estimate the rates of OS and disease-free survival (DFS), and the log-rank test was utilized to compare OS rates. Univariate or multivariate analysis was performed using the Cox model. Finally, the nomogram was established by using the regression modeling strategies package according to the results of multivariate analysis and clinical value. Propensity-score matching (PSM) analysis was applied to reduce patient selection bias. Propensity scores were calculated within each cohort using multivariate logistic regression models. Propensity scores included covariates that might affect both the likelihood of patients to receive the treatment of interest and the outcome of interest; they were unbalanced between treatment groups before matching. These variables included a number of patient characteristics as well as markers of disease severity. *p* < 0.05 was defined as statistically significance.

## Results

A total of 129 patients were included in this study (TACE, 78; LR, 51). Patients’ mean age was 50.88 ± 13.35 and 51.78 ± 12.89 years in the TACE and LR groups, respectively. The median tumor size was 4.21 and 3.8 cm in the TACE and LR groups, respectively. Additional baseline characteristics of pre- and post-PSM are summarized in Table 1.

**Table 1:** Baseline characters before and after PSM

Variables	The entire cohort			The PSM cohort		
	TACE (N = 78)	Surgery (N = 51)	*p* value	TACE (N = 31)	Surgery (N = 31)	*p* value
Sex			0.624			1.000
Female	11 (14.1%)	9 (17.6%)		6 (19.4%)	5 (16.1%)	
Male	67 (85.9%)	42 (82.4%)		25 (80.6%)	26 (83.9%)	
Age level			1.000			1.000
<60	57 (73.1%)	37 (72.5%)		25 (80.6%)	24 (77.4%)	
≥60	21 (26.9%)	14 (27.5%)		6 (19.4%)	7 (22.6%)	
Age (years)	50.88 ± 13.35	51.78 ± 12.89	0.705	52.00 ± 10.37	52.00 ± 10.22	0.922
HBsAg			0.121			0.174
Negative	20 (25.6%)	20 (39.2%)		7 (22.6%)	13 (41.9%)	
Positive	58 (74.4%)	31 (60.8%)		24 (77.4%)	18 (58.1%)	
Anti-HCV			0.152			0.238
Negative	74 (94.9%)	51 (100.0%)		28 (90.3%)	31 (100.0%)	
Positive	4 (5.1%)	0 (0%)		3 (9.7%)	0 (0%)	
TBIL (umol/L)	22.50 ± 24.51	27.32 ± 70.23	0.580	22.67 ± 20.47	19.14 ± 21.34	0.512
ALT (IU/L)			0.443			0.293
<35	27 (34.5%)	14 (27.5%)		14 (45.2%)	9 (29.0%)	
≥35	51 (65.4%)	37 (72.5%)		17 (54.8%)	22 (71.0%)	
AST (IU/L)			0.422			0.786
<35	19 (24.4%)	16 (31.4%)		9 (29.0%)	11 (35.5%)	
≥35	59 (75.6%)	35 (68.6%)		22 (71.0%)	20 (64.5%)	
Albumin (g/L)	40.32 ± 5.85	41.79 ± 5.97	0.169	39.78 ± 6.39	41.80 ± 4.69	0.162
APTT	30.71 ± 6.16	28.87 ± 4.04	0.065	31.45 ± 5.68	28.53 ± 3.33	0.016
INR	1.26 ± 1.40	1.05 ± 0.17	0.276	1.50 ± 2.19	1.05 ± 0.19	0.261
AFP (<400/≥400 ug/L)			1.000			1.000
<400	46 (59.0%)	30 (58.8%)		22 (71.0%)	21 (67.7%)	
≥400	32 (41.0%)	21 (41.2%)		9 (29.0%)	10 (32.3%)	
Platelet count (×10^^9^/L)			0.340			1.000
<100	29 (37.2%)	14 (27.5%)		10 (32.3%)	10 (32.3%)	
≥100	49 (62.8%)	37 (72.5%)		21 (67.7%)	21 (67.7%)	
CNLC stage			0.092			1.000
IIa	31 (39.7%)	28 (54.9%)		16 (51.6%)	16 (51.6%)	
IIb	47 (60.3%)	23 (45.1%)		15 (48.4%)	15 (48.4%)	
Ascites			0.362			0.425
NO	69 (88.5%)	48 (94.1%)		26 (83.9%)	29 (93.5%)	
YES	9 (11.5%)	3 (9.3%)		5 (16.1%)	2 (6.5%)	
Largest tumor size (<5/≥5 cm)			0.629			1.000
<5	64 (82.1%)	44 (86.3%)		26 (83.9%)	27 (87.1%)	
≥5	14 (17.9%)	7 (13.7%)		5 (16.1%)	4 (12.9%)	
Spiegel			1.000			1.000
NO	37 (47.4%)	25 (49.0%)		15 (48.4%)	16 (51.6%)	
YES	41 (52.6%)	26 (51.0%)		16 (51.6%)	15 (48.4%)	
Paracava			0.851			0.780
NO	52 (66.7%)	33 (64.7%)		23 (74.2%)	21 (67.7%)	
YES	26 (33.3%)	18 (35.3%)		8 (25.8%)	10 (32.3%)	
Caudate			0.112			0.570
NO	67 (85.9%)	38 (74.5%)		24 (77.4%)	21 (67.7%)	
YES	11 (14.1%)	13 (25.5%)		7 (22.6%)	10 (32.3%)	
Child-Pugh			0.147			1.000
A	61 (78.2%)	34 (66.7%)		23 (74.2%)	23 (74.2%)	
B	17 (21.8%)	17 (33.3%)		8 (25.8%)	8 (25.8%)	
ECOG 1	78 (100.0%)	51 (100.0%)		31 (100.0%)	31 (100.0%)	

Note: PSM Propensity score matching, HBsAg Hepatitis B surface antigen, HCV Hepatitis C virus, TBIL Total bilirubin, ALT Glutamic-pyruvic transaminase, AST Glutamic oxalacetic transaminase, APTT Activated partial thromboplastin time, INR International normalized ratio, AFP Alpha-fetoprotein, CNLC China liver cancer staging, ECOG Eastern cooperative oncology group.

### Overall survival

**Figure 1:**
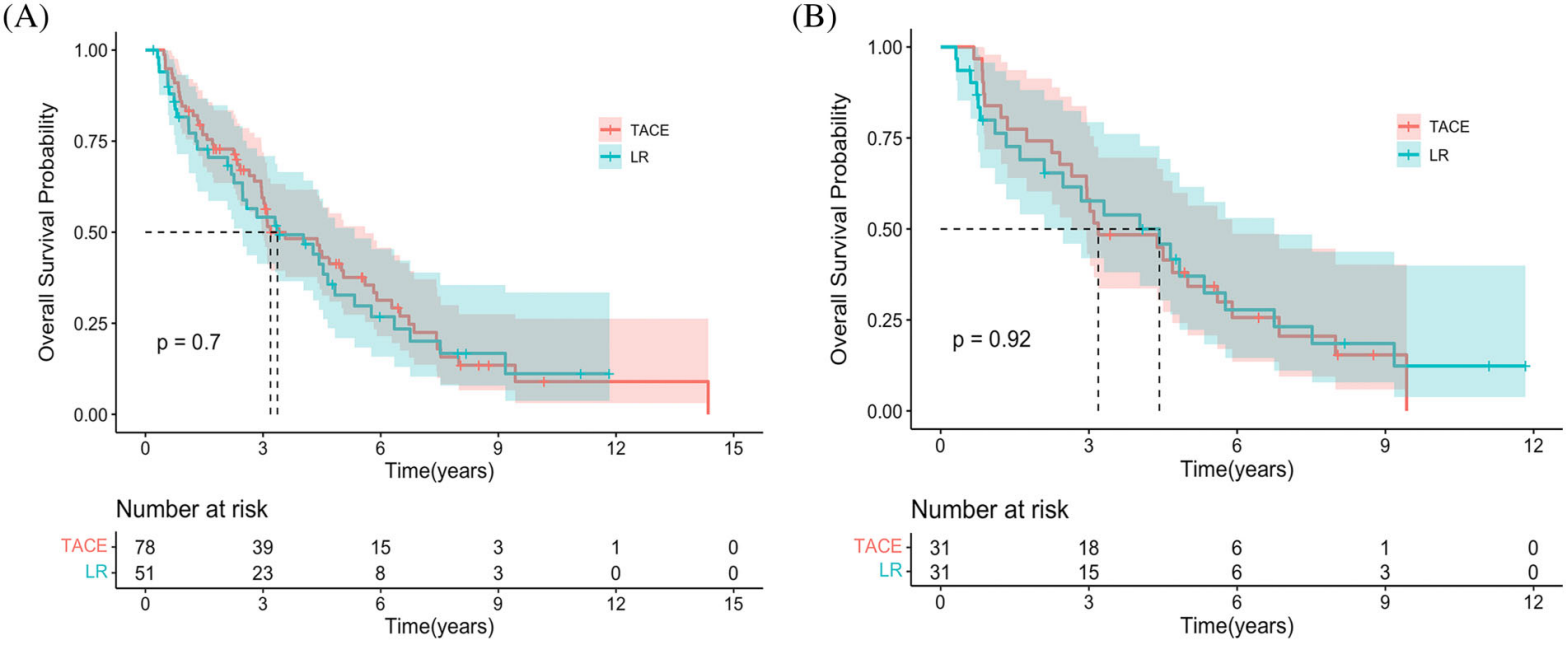
Kaplan-Meier curves demonstrating overall survival in both groups. (A) Overall survival curves of the entire cohort. (B) The overall survival curves of the propensity score–matching cohort.

The median OS was 3.19 years (95% CI, 2.97–5.0) and 3.37 years (95% CI, 2.48–5.33) in the TACE and LR groups, respectively (HR 0.92 [95% CI, 0.60−0.41; *p* = 0.706]) (Fig. 1A). Additionally, the estimated OS rates at 6 months and 1, 2, 3, 4, and 5 years were 94.9% *vs*. 86.3%, 84.6% *vs*. 72.5%, 66.7% *vs*. 60.8%, 50.0% *vs*. 43.1%, 35.9% *vs*. 37.3%, and 28.2% *vs*. 21.6% in the TACE and LR groups, respectively (Table 2). After PSM, the median OS time was 4.43 years (95% CI, 2.48–6.75) and 3.19 years (95% CI, 2.65–5.90) in the TACE and LR groups, respectively (HR 1.03 [95% CI, 0.58−1.83; *p* = 0.923]) (Fig.1B). The estimated OS rates at 6 months and 1, 2, 3, 4, and 5 years were 100% *vs*. 96.8%, 83.9% *vs*. 71.0%, 74.2% *vs*. 61.3%, 58.1% *vs*. 48.4%, 45.2% *vs*. 45.2%, and 32.3% *vs*. 25.0% in the TACE and LR groups, respectively (Table 2).

**Table 2:** The overall survival rate before and after PSM

	TACE (N = 78)	Surgery (N = 51)	OR (95% CI)	*p*
**The entire cohort**				
6-months OS rate	74 (94.9)	44 (86.3)	0.99 (0.63–1.56)	0.967
1-year OS rate	66 (84.6)	37 (72.5)	0.97 (0.60–1.58)	0.901
2-year OS rate	52 (66.7)	31 (60.8)	0.96 (0.56–1.66)	0.895
3-year OS rate	39 (50.0)	22 (43.1)	1.09 (0.56–2.10)	0.802
4-year OS rate	28 (35.9)	19 (37.3)	0.89 (0.43–1.84)	0.745
5-year OS rate	22 (28.2)	11 (21.6)	1.40 (0.54–3.65)	0.492
**The PSM cohort**				
6-months OS rate	31 (100.0)	30 (96.8)	1.13 (0.62–2.04)	0.688
1-year OS rate	26 (83.9)	22 (71.0)	1.15 (0.60–2.24)	0.672
2-year OS rate	23 (74.2)	19 (61.3)	1.24 (0.60–2.57)	0.566
3-year OS rate	18 (58.1)	15 (48.4)	1.22 (0.52–2.84)	0.651
4-year OS rate	14 (45.2)	14 (45.2)	1.03 (0.41–2.63)	0.945
5-year OS rate	10 (32.3)	8 (25.0)	1.38 (0.41–4.63)	0.598

Note: PSM, Propensity score matching, OS, Overall survival.

### Subgroup analysis of overall survival before and after the propensity score–matching cohort

**Figure 2:**
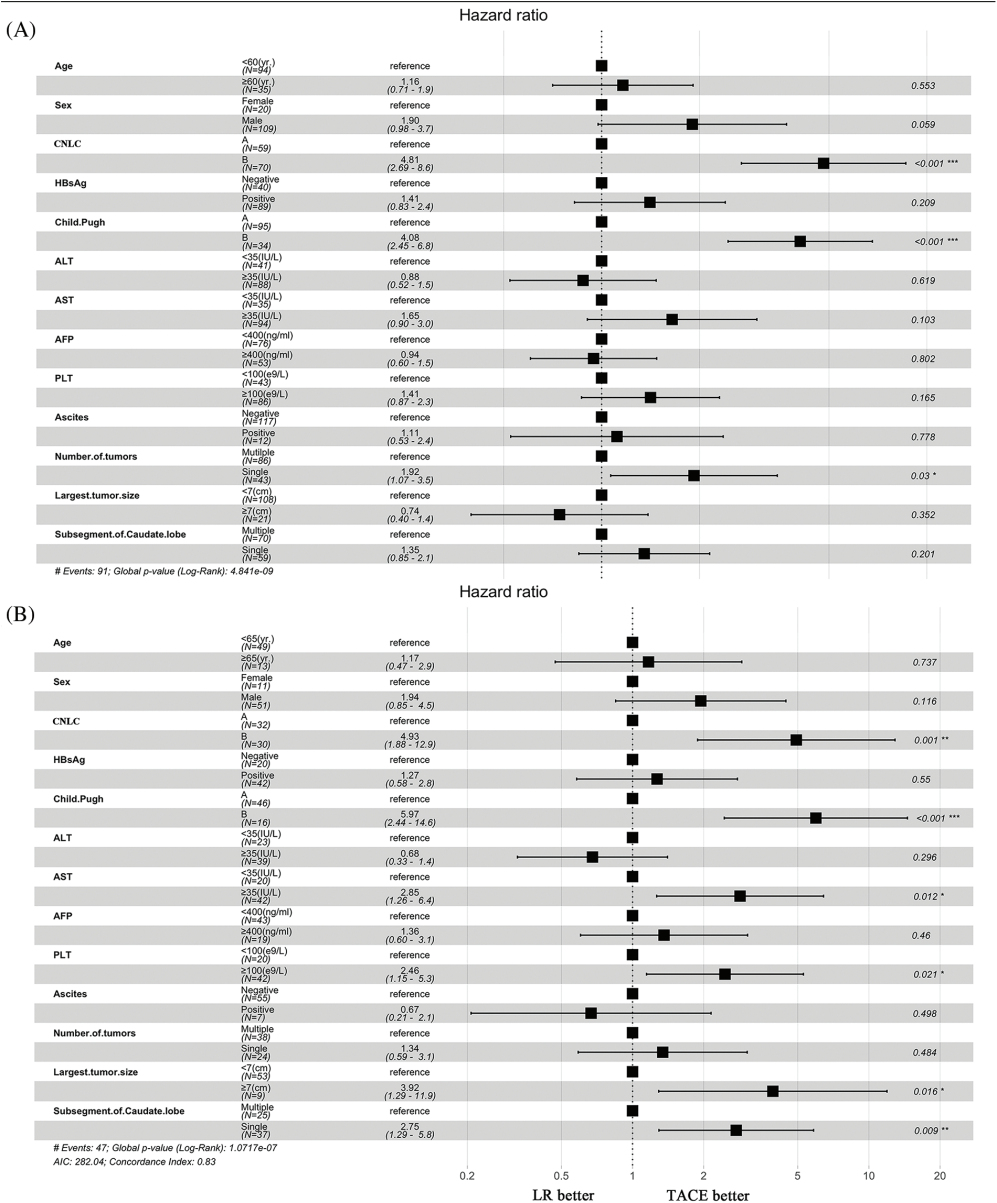
Subgroup analyses of overall survival. (A) Before propensity score matching. (B) After propensity score matching.

**Supplementary Figure S2:**
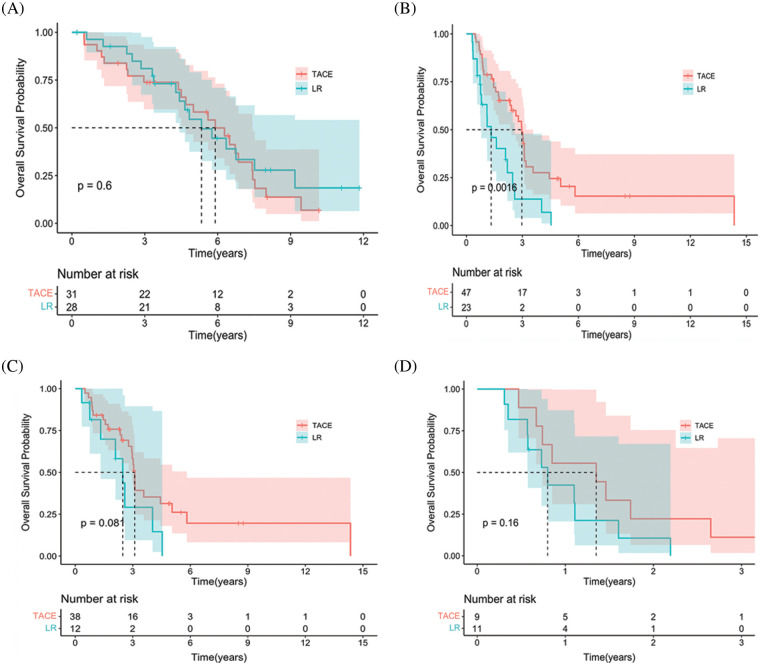
Subgroup Kaplan-Meier curves showing overall survival in the two groups. (A) Stage IIa Chinese liver cancer patients in the entire cohort. (B) Stage IIb Chinese liver cancer patients in the propensity score–matching cohort. (C) Child-Pugh A patients in the entire cohort. (D) Child-Pugh B patients in the propensity score–matching cohort.

For patients in stage CNLC-IIb, TACE had a longer OS than LR in the entire cohort (*p* = 0.002). Interestingly, there was no difference in stage CNLC-IIa HCC between TACE and LR (*p* = 0.6). Based on Child-Pugh A and B, TACE had a tendency toward longer OS compared with LR (*p* = 0.081) and (*p* = 0.16), respectively (Suppl. Figs. S2A–S2D). After PSM, TACE was associated with a longer OS in terms of stage CNLC-IIb (HR 4.93 [95% CI, 1.88−12.9; *p* = 0.001]), Child-Pugh B (HR 5.97 [95% CI, 2.44−14.6; *p* < 0.001]), AST (>35 IU/L; HR 5.97 [95% CI: 2.44−14.6; *p* < 0.001]), PLT (≥100 × 10^9; HR 2.46 [95% CI, 1.15−5.3; *p* = 0.021]) largest tumor size (≥5 cm; HR 3.92; [95% CI, 1.29–11.9; *p* = 0.016]) and single lesion (HR 2.75; [95% CI, 1.29–5.8; *p* = 0.009]), respectively (Figs. 2A and 2B).

### Univariate and multivariate analyses of overall survival in the propensity score–matching cohort

We performed additional univariate and multivariate Cox regression analyses with robust estimators in the total and PSM cohorts (Table 3). The following were found in the entire cohort: Child-Pugh score (HR 4.06 [95% CI: 2.13−7.13; *p* < 0.001]), CNLC stage (HR 0.32 [95% CI, 1.89−5.85; *p* < 0.001]), ascites (HR 1.51 [95% CI, 0.58−3.85; *p* = 0.391]), AFP (HR 1.39 [95% CI, 0.84−2.34; *p* = 0.211]), caudate (HR 1.38 [95% CI, 0.72−2.63; *p* = 0.332]), and anti-HCV (HR 2.59 [95% CI, 0.64−10.45; *p* = 0.18]), respectively. In the PSM cohort, the multivariate analysis including Child-Pugh score yielded the following: (HR 3.83 [95% CI, 1.57−9.31; *p* = 0.003]), CNLC stage (HR 1.39 [95% CI, 0.31−6.17; *p* = 0.668]), ascites (HR 1.16 [95% CI, 0.27−4.93; *p* = 0.843]), AFP (HR 1.65 [95% CI, 0.71−3.81; *p* = 0.241]), largest tumor size (HR 1.39 [95% CI, 0.31−6.17; *p* = 0.668]), and anti-HCV (HR 3.48 [95% CI, 0.60−20.30; *p* = 0.165]), respectively (Table 3).

**Table 3:** Univariate and multivariate analysis of OS

	The entire cohort						The PSMcohort					
Risk factor	Univariate			Multivariate			Univariate			Multivariate		
	HR	95% CI	*p* value	HR	95% CI	*p* value	HR	95% CI	*p* value	HR	95% CI	*p* value
Sex Female/Male	1.80	0.92–3.54	0.088				1.68	0.73–3.85	0.224			
Age yr.	1.01	0.99–1.02	0.342				1.02	0.98–1.05	0.354			
Age level <60/≥60	1.19	0.71–2.00	0.510				1.52	0.72–3.19	0.268			
HBsAg (Negative/Positive)	1.44	0.87–2.40	0.159				1.38	0.70–2.71	0.354			
ALT (<35/≥35 IU/L)	1.09	0.66–1.80	0.746				1.19	0.61–2.34	0.607			
AST (<35/≥35 IU/L)	1.28	0.74–2.21	0.379				1.63	0.79–3.37	0.190			
PLT (<100/≥100 × 10^^9^/L)	1.21	0.72–2.00	0.473				1.44	0.69–3.00	0.329			
Caudate lobe												
Largest tumor size (<5/≥5 cm)	1.22	0.62–2.40	0.562				4.09	1.31–12.71	0.015	1.39	0.31–6.17	0.668
Number of tumors (Single/Multipl)	1.01	0.63–1.62	0.981				0.68	0.35–1.34	0.264			
Spiegel (no/yes)	0.92	0.58–1.50	0.741				1.02	0.54–1.94	0.953			
Caudate (no/yes)	1.68	0.95–2.94	0.072	1.38	0.72–2.63	0.332	1.63	0.78–3.40	0.194			
Paracava (no/yes)	0.84	0.51–1.38	0.488				0.66	0.32–1.34	0.246			
ECOG	1.00	–	–				1.00	–	–			
APTT	1.05	1.01–1.09	0.017				1.05	0.98–1.11	0.159			
Number of tumors	1.07	0.69–1.68	0.764				1.10	0.62–1.94	0.753			
TBIL (umol/L)	1.00	1.00–1.01	0.835				0.99	0.98–1.01	0.460			
INR	1.17	1.00–1.37	0.052				1.15	0.97–1.37	0.110			
Serum albumin level (g/L)	1.00	0.95–1.04	0.857				0.99	0.92–1.06	0.742			
Child-Pugh (A/B)	4.32	2.58–7.23	<0.001	4.06	2.31–7.13	<0.001	6.62	2.96–14.81	<0.001	3.83	1.57–9.31	0.003
CNLC stage (IIa/IIb)	3.11	1.85–5.21	<0.001	0.32	1.89–5.85	<0.001	7.31	3.19–16.79	<0.001	5.12	2.07–12.66	<0.001
Ascites (no/yes)	4.76	2.19–10.36	<0.001	1.51	0.59–3.85	0.391	3.51	1.19–10.42	0.023	1.16	0.27–4.93	0.843
AFP (<400/≥400 ug/L)	1.57	0.96–2.57	0.072	1.39	0.83–2.34	0.211	2.04	0.96–4.33	0.065	1.65	0.71–3.81	0.241
Anti-HCV (Negative/Positive)	4.21	1.27–13.94	0.018	2.59	0.64–10.45	0.18	4.5	1.26–16.05	0.02	3.48	0.60–20.30	0.165

Note: PSM Propensity score matching, HR Hazard ratio, CI Confidence interval, PLT Platelets, HBsAg Hepatitis B surface antigen, HCV, Hepatitis C virus, TBIL Total bilirubin, ALT Glutamic-pyruvic transaminase, AST Glutamic oxalacetic transaminase, APTT Activated partial thromboplastin time, INR International normalized ratio, AFP Alpha-fetoprotein, CNLC China liver cancer staging, ECOG Eastern cooperative oncology group.

### Prognostic nomogram for overall survival

**Figure 3:**
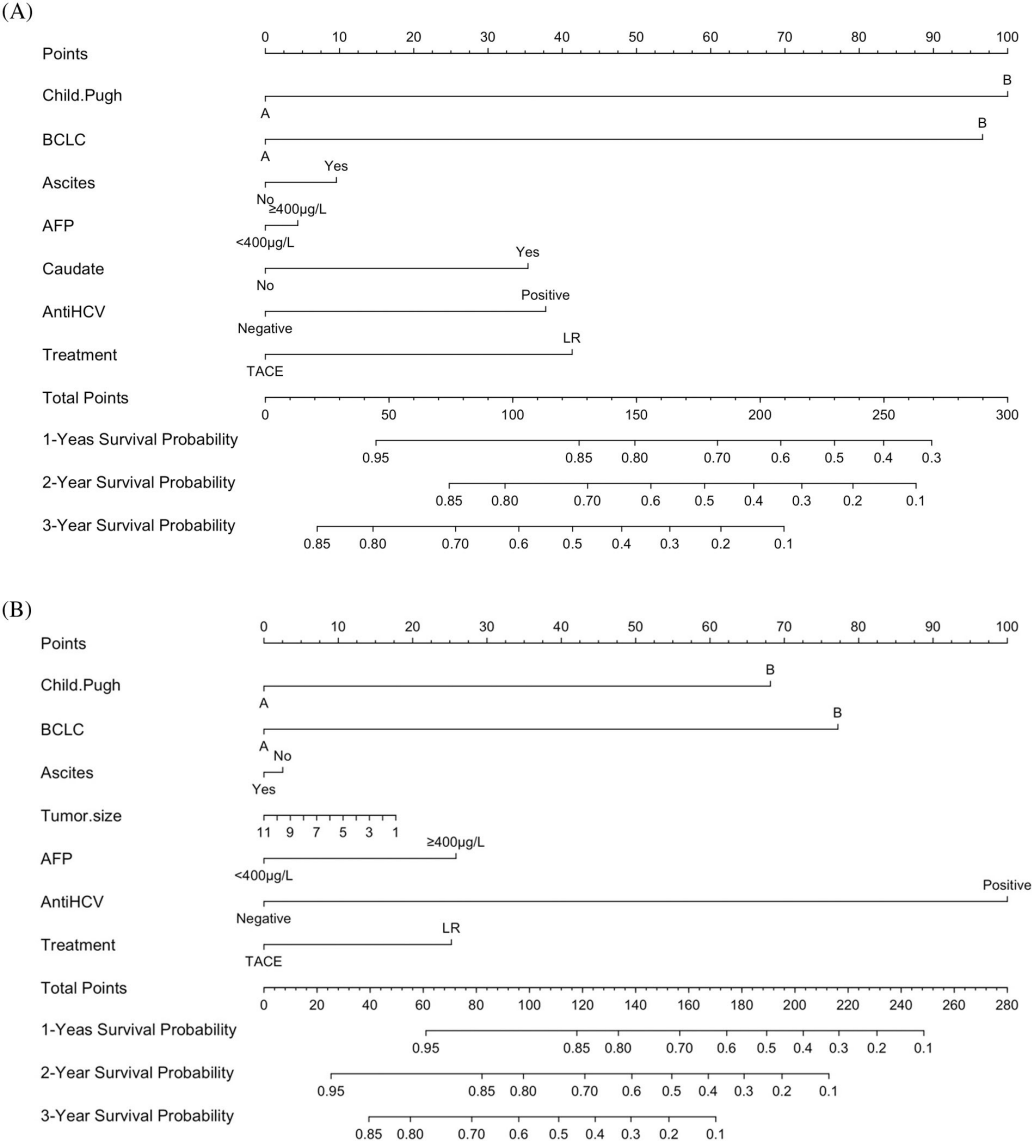
Development of predictive nomograms for both groups. (A) Predictive nomograms for the entire cohort. (B) Predictive nomograms for the propensity score–matching cohort.

Independent prognostic factors based on the multivariate analysis and clinical values for the two groups were determined to establish predictive nomograms. For survival estimation of HCC, the factors were Child-Pugh (A/B), CNLC (IIa/IIb), ascites (no/yes), AFP (<400/≥400 μg/L), caudate (no/yes), anti-HCV (negative/positive), and treatment (TACE/LR) (Fig. 3A). After PSM, the predictive nomogram included Child-Pugh score (A/B), CNLC (IIa/IIb), ascites (no/yes), AFP (<400/≥ 400 μg/L), tumor size (<5/≥5 cm), anti-HCV (negative/positive), and treatment (TACE/LR) (Fig. 3B). Additionally, the calibration curves showed good agreement between prediction and observation in the probability of 1- and 2-year survival.

### Adverse events

No hospital mortalities were observed in either group. However, four patients died of incurable hepatic failure within 30 days after LR. One patient developed a pancreatic fistula after LR and was successfully treated by abdominal drainage. Another patient died of sepsis within 30 days after TACE. The most common adverse events following TACE were transient fever (*n* = 60), transient abdominal pain (*n* = 42), nausea and vomiting (*n* = 18), and fatigue (*n* = 14). No complications were observed in patients with CTCAE of grade 4 or higher in either group.

## Discussion

HCC originating in the caudate lobe tends to involve adjacent major portal veins, hepatic veins, and/or the inferior vena cava. Hence, HCC occurring exclusively in the caudate lobe and originating in the caudate lobe presents a therapeutic challenge and has a poor OS [2]. To the best of our knowledge, this is the first study comparing TACE with LR and the largest study to evaluate the clinical outcomes of TACE treatment for HCC occurring exclusively in the caudate lobe.

Previously, limited evidence presented TACE as effective for treating HCC in the caudate lobe. Woo et al. [22] reported that TACE treatment for HCC originating in the caudate lobe led to a complete response in 51.4% of their patients, a partial response in 25.7%, stable disease in 0.09%, and progression of disease in 0.09%. Kim et al. [3] reported that OS rates among their TACE-treated patients at 1, 2, 3, 4, and 5 years were 92%, 79%, 65%, 56%, and 56%, respectively. Other studies have reported OS rates at 1, 2, 3, 4, and 5 years of 71.7%, 58.9%, 41.0%, 30.5%, and 28.3%, respectively [15–17,19]. DFS rates at 6, 12, 18, and 24 months were 61.7%, 44.0%, 22.0% and 20.3% respectively. The OS and DFS rates of our patients, falling in the median range, are consistent with those of previous reports [15–17,19]. However, Kim et al. [3] found that portal vein thrombosis caused a shorter OS. Notably, these studies involved different research periods. The study of Kim et al. [3] evaluated data collected from 1998 to 2009, and our institution has used superselective chemoembolization treatment since 2004. Our study excluded portal vein tumor thrombosis (PVTT) patients because Vp3 or Vp4 PVTT is a contraindication for LR [23]. Additionally, Vp1/Vp2 PVTT complicates accurate diagnosis. In our study, Child-Pugh (A/B), CNLC (IIa/IIb), ascites (no/yes), AFP (<400/≥400 μg/L), caudate (no/yes), anti-HCV (negative/positive) are independent factors.

Previously, Tanaka et al. [2] presented data indicating that 15 patients with HCC originating in the caudate lobe had received LR treatment. Intrahepatic recurrence (40%) was higher among these patients than among those with HCC in other lobes (17.6%; *p* < 0.05). Sakamoto et al. [24] reported that 46 patients with HCC originating in the caudate lobe had lower OS than those with HCC in other lobes (45% *vs*. 76%). Similarly, another study found that the 5-year OS after resection was 25.9%, significantly lower than of disease involving other lobes (54.1%; *p* = 0.01) [2]. These results indicate that LR for HCC originating in the caudate lobe continues to pose a challenge. Zhou et al. [17] reported that 23 patients who received caudectomy achieved OS benefits at 1, 3, and 5 years of 90.1%, 60%, and 37%, respectively. Liu et al. [19] reported that patients with HCC originating in the caudate lobe who received hepatectomy achieved 1-, 3-, and 5-year DFS of 65.7%, 38.1%, and 18.4% and OS of 76.1%, 54.7%, and 31.8%, respectively. Another study [25] demonstrated that patients (*n* = 30) treated with isolated caudate lobe resection achieved 1-, 3-, and 5-year OS of 62%, 34%, and 11%, respectively and 3-year DFS of 31%. Wang et al. [7] described 43 cases of HCC in the caudate lobe where patients achieved the 1-, 3-, and 5-year OS rates of 83.5%, 66.5%, and 29.3% and 1-, 3-, and 5-year DFS of 66.7%, 40.4%, and 20.2%, respectively. The critical differences among these studies [25,17,7] may stem from their small sample sizes and differing inclusion criteria.

We first indicated that TACE has a tendency to surpass LR in 1-, 3-, and 5-year OS rates. The Cox model revealed that OS is related to Child-Pugh scores, CNLC stage, ascites, AFP, caudate, and anti-HCV. Our results are similar to those reported by Liu et al. [19], who performed a multivariate analysis revealing significant independent prognostic factors, including subsegmental liver cirrhosis, location of the tumor, and surgical margin. Moreover, we found that CNLC-IIb stage, Child-Pugh B, AST (>35 IU/L), PLT 100 × 10^9, largest tumor size (≥5 cm), and tumor location within a single subsegment of the caudate lobe resulted in a better OS in the TACE group. Interestingly, according to the CNLC guideline, LR was better than TACE at treating HCC at stage CNLC-IIa, which conflicts with the results of our study; our data indicated no difference between LR and TACE when HCC occurred in the caudate lobe. The inconsistency of this result may stem from the relatively small sample size of our study and the complex feeding arteries of HCC in the caudate lobe. For stage CNLC-IIb HCC in the caudate lobe, TACE had a better OS than LR. Similar results were found in Child-Pugh A/B patients. Both TACE and LR are recommended for the initial treatment of patients with HCC at the stage CNLC-IIb according to the guidelines for the diagnosis and treatment of primary liver cancer in China [15]. Although TACE may bring more benefit than LR. LR was recommended as an alternative treatment for patients who could not receive TACE. In this regard, a stricter preoperative multidisciplinary assessment is required [15]. Similarly, TACE treatment was superior to LR in patients with BCLC-B stage [13]. Combined with our research data, it is shown that patients with CNLC-IIb stage or BCLC-B stage HCC may benefit more from TACE than LR regardless of whether the tumor is located in the caudate lobe or another hepatic lobe.

The most common adverse event noted In our study was postembolization syndrome, including fever, nausea, vomiting, and right-upper-quadrant abdominal pain. Most of the complications were self-limited, and there were no procedure-related deaths. Another study [25] reported that 30 patients treated by isolated caudate lobe resection had a hospital morbidity rate of 33% without postoperative mortality. However, more patients in the LR group died of hepatic failure, which indicates that LR may pose a higher risk of hepatic damage in the long term. TACE was associated with more frequent transient complications than LR; hence the short-term complications warrant consideration.

Our study has several limitations. First, this was a retrospective study with a relatively small sample size. Second, instead of choosing all boundaries as mean or median values, the cutoffs should be more carefully evaluated. Third, the efficacy of nomogram may not be accessed sufficiently and effectively without external validation in other cohorts. Last, our study focused on the survival of patients with caudal lobe HCC and the factors that affected their survival, but the possible changes in the characteristics of caudate lobe HCC intervention therapy, such as the tumor-feeding vessel, had limited consideration. Our study’s major strength lies in its case numbers, which were somewhat larger than those of similar studies. Therefore our survival statistics may better reflect the clinical reality.

## Conclusion

Based on this study, treatment with TACE may be associated with a longer OS than liver resection in patients with CNLC-IIb HCC of the caudate lobe. Owing to the limitations of the present study’s design and its still relatively small sample size, additional randomized controlled trials will be needed to confirm its findings.

## Data Availability

The data analysed during the current study are available from the corresponding author on reasonable request.
